# Synergistic Effects of Aldehyde Dehydrogenase 2 Polymorphisms and Alcohol Consumption on Cognitive Impairment after Ischemic Stroke in Han Chinese

**DOI:** 10.1155/2021/6696806

**Published:** 2021-06-24

**Authors:** Ying Yu, Jie Gao, Shasha Wang, Heng Lv, Liping Xiao, Hengyuan Shi, Xianjie Jia

**Affiliations:** ^1^Department of Physiology, School of Basic Medicine, Bengbu Medical College, Bengbu 233000, China; ^2^Department of Physiology and Pathophysiology, School of Basic Medicine, Shandong University, Jinan 250012, China; ^3^Key Laboratory of Cardiovascular and Cerebrovascular Diseases, Bengbu Medical College, Bengbu 233030, China; ^4^Department of Epidemiology and Statistics, School of Public Health, Bengbu Medical College, Bengbu 233000, China

## Abstract

Aldehyde dehydrogenase 2 (*ALDH2*) polymorphisms are related to both stroke risk and alcohol consumption. However, the influence of *ALDH2* polymorphisms and alcohol consumption on cognitive impairment after ischemic stroke remains unknown, as do the possible mechanisms. We enrolled 180 Han Chinese ischemic stroke patients from four community health centers in Bengbu, China. Cognitive function was assessed using the Montreal Cognitive Assessment (MoCA), and two different MoCA cutoff scores were used to define cognitive impairment in ischemic stroke patients. The *ALDH2* genotypes were determined using polymerase chain reaction and direct sequencing. To assess the associations of *ALDH2* polymorphisms and alcohol consumption with cognitive impairment after ischemic stroke, we performed binary logistic regression analysis with odds ratios. We revealed that individuals with the *ALDH2* wild-type genotype were more likely to have high MoCA scores than those with the mutant and heterozygous types (*p* = 0.034). In addition, using two MoCA cutoff scores, the percentage of moderate to excessive alcohol consumption in the cognitive impairment group was higher than that in the nonimpairment group (*p* = 0.001). The levels of 4-hydroxy-2-nonenal (*p* = 0.001) and swallowing function (*p* = 0.001) were also higher in the cognitive impairment group than in the nonimpairment group. Moreover, after adjusting for other potential risk factors, *ALDH2* polymorphisms and alcohol consumption had a significant synergistic effect on cognitive impairment (*p* = 0.022). Specifically, the *ALDH2*∗*2* mutant allele and higher alcohol consumption were associated with cognitive impairment and swallowing ability after ischemic stroke. Targeting ALDH2 may be a useful biomarker for cognitive rehabilitation following ischemic stroke.

## 1. Introduction

Stroke is an important problem in public health and is the leading cause of adult disability worldwide [1, 2]. It has been reported that approximately 15 million people per year have a stroke [3]. Most patients experience some disturbance in cognitive function following stroke [4], and cognitive function is a significant focus in stroke rehabilitation [5]. Alcohol consumption, which is common worldwide, affects the development of stroke and cognitive performance [6, 7]. Epidemiological evidence has revealed that excessive drinking is a major risk factor for all stroke subtypes, but especially for ischemic stroke [8, 9]. Additionally, some cohort studies have suggested that light to moderate drinking may have a protective effect on cardiovascular disease and ischemic stroke [9, 10]. In contrast, more recent studies have indicated that alcohol consumption is roughly linearly associated with stroke risk [11, 12]. However, a U-shaped relationship has also been reported between regular alcohol consumption and cognitive function in several major epidemiological studies [13]; thus, the relationship between alcohol consumption and cognitive impairment after ischemic stroke remains uncertain.

Alcohol metabolism represents a key biological determinant that can impact drinking behavior. Aldehyde dehydrogenase 2 (ALDH2) is the primary enzyme involved in this metabolic process [14]. Previous research has examined the potential effects of ALDH2 on alcohol consumption and health outcomes. However, the relationship between ALDH2, alcohol consumption, and cognitive function in patients with ischemic stroke is unclear. The main function of ALDH2 is to detoxify acetaldehyde, which is a toxic chemical product of ethanol metabolism [15]. Moreover, ALDH2 also removes other toxic aldehydes, such as 4-hydroxy-2-nonenal (4-HNE). As a potential substrate of ALDH2, 4-HNE is commonly considered a specific marker of ischemic stroke injury [15, 16].

ALDH2 is abundant in the brain, heart, lungs, and other organs with high mitochondrial contents [17, 18]. The *ALDH2* gene consists of 13 exons and 12 introns. In exon 12, a polymorphism exists, in the form of a G-to-A missense mutation. The glutamate at position 504 is substituted by lysine (Glu504Lys). This polymorphism is also known as rs671, or the *ALDH2*∗*2* form, while the more common wild-type form is known as *ALDH2*∗*1*. There are thus three possible allele combinations in the population: wild-type (∗1/∗1), heterozygote (∗1/∗2), and mutant (∗2/∗2) [19, 20]. Approximately 40% of the East Asian population carries an *ALDH2*∗*2* mutant allele, with a resulting marked reduction in enzymatic activity [15]. Recent studies have reported that the Glu504Lys polymorphism may affect ischemic stroke risk in the Han Chinese population and that carriers of the *ALDH2*∗*2* allele have increased 4-HNE levels after stroke [21]. However, there has been little previous research into the association between *ALDH2* genotypes and cognitive impairment after ischemic stroke.

Therefore, the aim of this study was to evaluate the association of *ALDH2* genotypes and alcohol consumption with cognitive function after ischemic stroke. Cognitive function can be tested briefly using the Montreal Cognitive Assessment (MoCA), and this test is recommended for screening cognitive impairment in patients with ischemic stroke [22]. In many previous studies, cognitive impairment has been defined as a MoCA score < 26 [23]. However, some researchers have recommended that a MoCA cutoff score of 22/23 points might be more suitable for detecting cognitive impairment [24]. In addition, swallowing deficits are also commonly reported in patients with ischemic stroke [25], and cognitive dysfunction is related to dysphagia [26]. In the present study, we used these two different MoCA cutoff scores to investigate the relationship between alcohol consumption, 4-HNE levels, swallowing function, and cognitive impairment after ischemic stroke, respectively. We further investigated the synergistic effects of *ALDH2* genotype and alcohol consumption on the MoCA score and swallowing ability in ischemic stroke patients. Finally, we sought to explore the underlying mechanisms that might influence these associations.

## 2. Methods

### 2.1. Patients

From June 2015 through August 2015, patients with ischemic stroke from four community health centers located in the Longzihu District of Bengbu (Anhui Province, China) were recruited in our study. We visited each community and held a free health checkup for participants. Each participant completed a self-reported questionnaire relating to their lifestyle and medical history, including information on prior stroke and baseline disease status. The inclusion criteria for ischemic stroke patients were as follows: (i) stroke diagnosis as per the revised diagnostic criteria of the 4th National Cerebrovascular Disease Conference in China [27], (ii) stroke diagnosis based on computed tomography or magnetic resonance imaging brain scans, (iii) within 3 months after stroke onset, (iv) permanent residents of Han Chinese ethnicity in selected communities, and (v) informed consent provided. The exclusion criteria were as follows: (i) previous history of cerebral vascular malformation, transient ischemic attack, intracranial hemorrhage, stroke mimics (i.e., seizures or migraines), or neurological deficits; (ii) previous history of bleeding diathesis, anticoagulation therapy, illicit drug use, or serious medical illness; (iii) previous history of illiteracy or any major mental or physical condition that may interfere with cognitive assessments; and (iv) a diagnosis of coronary artery disease [19, 22, 28].

**Figure 1:**
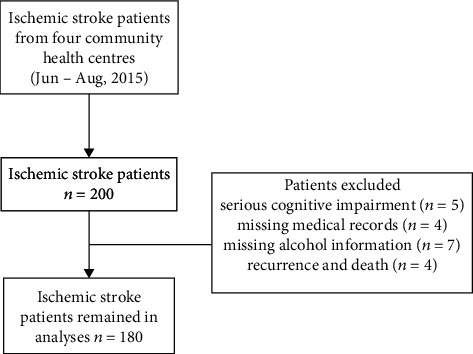
Flowchart of the participant selection process.

Data were initially obtained from 200 participants. We excluded patients who were unable to participate in the interview because of serious cognitive impairment (*n* = 5) and those with missing medical records (*n* = 4). We also excluded subjects who had missing information regarding alcohol habits, such as alcohol status and the amount and frequency of alcohol consumption (*n* = 7), and regarding recurrence and death (*n* = 4). Thus, a total of 180 patients with ischemic stroke were enrolled in this study. A detailed flowchart showing participant selection is provided in Figure 1.

### 2.2. Demographic and Clinical Characteristics and Measurements

We obtained patient demographic information from patients' medical charts and self-reported data. According to the medical charts, patients were divided into two subtypes using a simple clinical scheme with the Oxfordshire Community Stroke Project (OCSP) classification and included the following: posterior circulation infarction (PCI) and anterior circulation infarction (ACI) [29]. In the PCI group, the infarcts involved the brainstem, posterior cerebral artery area, thalamus, or cerebellum; in the ACI group, the infarcts occurred in the region of the middle cerebral artery, anterior cerebral artery, or anterior choroidal artery [30].

After fasting for 8 to 12 hours, we measured each patient's blood pressure, height, and weight and calculated their body mass index (BMI). In addition, fasting venous blood samples were collected at approximately the same time of day, in the morning, to minimize diurnal variations [31]. Each blood sample was drawn into a tube containing ethylenediaminetetraacetic acid as an anticoagulant and a tube without anticoagulant. The obtained plasma and serum were preserved at -80°C until assays were performed. Routine blood and biochemistry tests were analyzed, including fasting plasma glucose (FPG), total cholesterol (TC), triglycerides (TG), high-density lipoprotein cholesterol (HDL-C), and low-density lipoprotein cholesterol (LDL-C). This part of the study was approved by the ethics committee of Bengbu Medical College. The cognitive assessments were conducted at the same time as the blood samples were taken.

### 2.3. Cognitive Assessments

The MoCA is a cognitive screening tool that can be used to distinguish healthy cognitive aging from mild cognitive impairment [32]. It is simple to conduct, sensitive, and valid. Since its introduction into clinical practice, it has been repeatedly demonstrated to be suitable for the initial assessment of mental status and for follow-up assessments [33]. The MoCA was administered by trained physicians in each community.

The MoCA comprises eight subtests that involve visuospatial/executive, naming, memory, attention, language, abstraction, delayed recall, and orientation with respect to time and place. MoCA scores range from 0 to 30. A higher score indicates better cognitive performance [34, 35]. With a cutoff of 26 (which we used as Method 1 in our study), the sensitivity and specificity of MoCA have been reported as 90% and 87%, respectively, when administered to screen patients with mild cognitive impairment in Canada [32]. However, subsequent clinical studies have demonstrated that some patients with normal cognitive ability have MoCA scores below 26 [36]. The MoCA cutoff scores of ischemic stroke patients by the educational level have been reported as follows: 24/25 for individuals with ≥7 years of education, 19/20 for individuals with 1 to 6 years of education, and 13/14 for illiterate individuals. Therefore, we also used a cutoff point of 22/23 (which we used as Method 2 in our study) for MoCA scores [24, 31, 37].

### 2.4. Water-Swallowing Test

The water-swallowing test (WST) is frequently used in clinical practice as a functional assessment to evaluate swallowing function [38]. Swallowing performance was assessed with the 30 mL water swallowing test which is cheap, easy to use, and with the highest reliability [39]. A total of 30 mL water was put on a plastic cup. The patient was ordered to drink the water “as quickly as comfortably possible” in an upright seated position. The time to drink and presence or absence of coughing were recorded. The results included the following five levels: level I (drink once, no coughing), level II (drinking more than two times of interruption, no coughing), level III (drinking once, with coughing), level IV (drinking more than two times of interruption, with coughing), and level V (coughing frequently and cannot drink the water successfully). After examination, swallowing ability was classified as normal (level I within 5 s), possible abnormality (level I over 5 s or level II), and abnormality (levels III to V). Possible abnormality and abnormality are considered dysphagia [39].

### 2.5. Alcohol Consumption Measurements

Data regarding alcohol consumption were collected via a self-administered questionnaire [40]. The questionnaire included a range of drinking variables in the past 12 months before the stroke. The type of alcohol (liquor, beer, or wine), quantity of consumption, and frequency of consumption (never or occasionally, daily, weekly, or monthly) were all assessed. The average daily intake of absolute alcohol was estimated based on the quantity and frequency of consumption. The content of ethanol (pure alcohol) was assumed to be 15.1 g for a drink of liquor, 13.2 g for a can of beer, and 10.8 g for a standard glass of wine [41, 42]. For each participant, total ethanol intake was converted to standard units per week (1 unit = 8 g ethanol). Each participant's drinking status was then classified as one of four distinct categories: nondrinker, light drinker, moderate drinker, or excessive drinker [41]. Nondrinkers consumed <1 unit of ethanol per week. Light drinkers consumed 1-10 units/week for men and 1-7 units/week for women. Moderate drinkers consumed 11-21 units/week for men and 8-14 units/week for women. Excessive drinkers consumed >21 units/week for men and >14 units/week for women.

### 2.6. 4-HNE Concentration Measurements

The plasma levels of 4-HNE were estimated using ELISA kits (Elabscience Biotechnology, Wuhan, China) according to the manufacturer's instructions [21].

### 2.7. ALDH2 Genotyping Measurements

Genomic DNA samples were obtained from blood samples using commercial DNA extraction kits (Tiangen Biotech, Beijing, China). The primer sequences were as follows: forward primer, 5′-GTCAACTGCTATGATGTGTTTGG-3′ and reverse primer, 5′-CCACCAGCAGACCCTCAAG-3′. The 50 *μ*L polymerase chain reaction (PCR) mixture consisted of 2 *μ*L DNA template, 2 *μ*L forward and 2 *μ*L reverse primers, 25 *μ*L TaqMan Master Mix, and 19 *μ*L double-distilled H_2_O. The PCR was conducted with predenaturation for 3 min at 94°C, followed by 35 cycles of amplification (94°C for 45 s, 53°C for 30 s, and 72°C for 45 s), and extension for 5 min at 72°C [20]. The PCR products were purified using commercial kits (Axygen Biosciences, Corning, NY, USA) and sent to GenScript Corporation (Nanjing, China) for sequencing.

### 2.8. Statistical Analyses

The results of continuous variables are presented as the mean ± standard deviation (SD), whereas the results of categorical variables are expressed as numbers of patients and percentages. Two-tailed Student's *t*-test or one-way ANOVA was performed for continuous variables, and the *χ*^2^ test was performed for categorical variables. Binary logistic regression analysis was performed to determine the associations of *ALDH2* polymorphisms and alcohol consumption with cognitive impairment and swallowing ability after ischemic stroke in a Han Chinese population by estimating the odds ratios (ORs) with 95% confidence intervals (CIs) [43]. We used two different cognitive impairment assessment methods (Method 1 and Method 2) to estimate the correlations between *ALDH2* polymorphism, alcohol consumption, and cognitive impairment. All missing values of predictors were imputed. Statistical analyses were conducted using SPSS version 24.0 software (IBM Corporation, Chicago, USA). All *p* values of less than 0.05 were taken as statistically significant.

## 3. Results

### 3.1. Baseline Characteristics

Table 1 shows the demographic characteristics of all participants, grouped by ALDH2 polymorphism. We combined the heterozygotes (ALDH2∗1/∗2) and mutant homozygotes (ALDH2∗2/∗2) into one category and compared them with the wild-type homozygotes (ALDH2∗1/∗1) in our analyses. There were no significant differences between the two categories in age or education. However, the levels of alcohol consumption were significantly higher in the ALDH2 wild-type genotype group than in the mutant and heterozygous genotype group (*p* = 0.006). We further compared alcohol consumption between two ALDH2 genotypes by gender; the results showed that there was no statistical difference between genotype and alcohol consumption in males. However, the levels of alcohol consumption were significantly higher in the ALDH2 wild-type genotype group than in the mutant and heterozygous genotype group in females (*p* = 0.001, Supplementary Table 1).

### 3.2. Clinical Characteristics

The clinical characteristics of participants according to ALDH2 polymorphism are shown in Table 2. There were no significant differences between the two groups in BMI, systolic blood pressure (SBP), diastolic blood pressure (DBP), or FPG, TG, HDL-C, or LDL-C levels. However, 4-HNE levels were higher in patients with mutant alleles (13.42 ± 2.11 ng/L) than in patients with wild-type alleles (12.18 ± 1.94 ng/L; *p* = 0.001), whereas TC levels were lower in patients with mutant alleles (5.62 ± 1.40 mmol/L) than in patients with wild-type alleles (6.16 ± 1.94 mmol/L; *p* = 0.037).

### 3.3. Analysis of Cognitive Performance in Subjects with Different ALDH2 Genotypes

**Figure 2:**
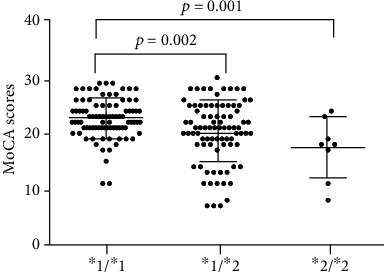
Comparison of Montreal Cognitive Assessment (MoCA) scores of ischemic stroke patients grouped by *ALDH2* genotype.

MoCA was used in ischemic stroke patients as a dependent variable to assess the extent of early vascular cognitive dysfunction. We compared the MoCA scores of three genotypes by analysis of variance (*F* = 8.643, *p* = 0.0003).  Figure 2 shows that the MoCA scores of the *ALDH2* wild-type genotype group (*n* = 87, 22.64 ± 3.55) were higher than those of the heterozygous group (*n* = 85, 20.28 ± 5.52; *p* = 0.001). The MoCA scores of the *ALDH2* wild-type genotype group (22.64 ± 3.55) were higher than that of the mutant genotype group (*n* = 8, 17.25 ± 5.45; *p* = 0.002). And there was no difference between the heterozygous and mutant genotype groups (*p* > 0.05) (Figure 2).

### 3.4. Association of Alcohol Consumption and 4-HNE Levels with Cognitive Impairment

We used two different cognitive impairment assessment methods to compare alcohol consumption between the cognitive impairment and nonimpairment groups (Table 3). For both of the MoCA cutoff scores, the percentage of moderate to excessive alcohol consumption was higher in the cognitive impairment group than in the nonimpairment group. According to the MoCA subscores of visuospatial/executive, naming, memory, attention, language, abstraction, delayed recall, and orientation, MoCA subscores were compared with alcohol consumption in two *ALDH2* genotypes. Intergroup differences were assessed using the single factor analysis of variance. Among the seven subscores, we found that two subscores (language and delayed recall) have a significant difference in alcohol consumption using univariate analysis (Supplementary Table 2).

**Figure 3:**
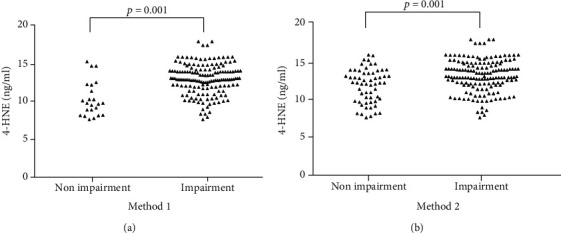
Comparison of 4-hydroxy-trans-2-nonenal (4-HNE) levels between the cognitive impairment and nonimpairment groups according to two different Montreal Cognitive Assessment (MoCA) cutoff scores: (a) score of 26; (b) score of 23.

We also used the two different MoCA cutoff scores to compare 4-HNE levels between the cognitive impairment and nonimpairment groups (Figure 3(a)). With a cutoff score of 26, the levels of 4-HNE were higher in the cognitive impairment group (12.94 ± 2.01, *n* = 157) than in the nonimpairment group (10.29 ± 2.29, *n* = 23). Furthermore, when we considered MoCA scores and educational levels of the ischemic stroke patients, with a cutoff score of 23 (Figure 3(b)), the levels of 4-HNE were also higher in the cognitive impairment group (12.98 ± 2.14, *n* = 121) than in the nonimpairment group (11.81 ± 2.22, *n* = 59).

### 3.5. Association between Swallowing Function and Cognitive Impairment

We used two different cognitive impairment assessment methods, to compare swallowing levels between the cognitive impairment and nonimpairment groups (Table 4). For both of the MoCA cutoff scores, the level of swallowing function was higher in the cognitive impairment group than in the nonimpairment group.

### 3.6. Separate Effects of ALDH2 Polymorphisms and Alcohol Consumption on Cognitive Impairment

To test the possible association of *ALDH2* polymorphism and alcohol consumption with cognitive impairment, we assessed the separate effects of *ALDH2* polymorphism and alcohol consumption on cognitive impairment in ischemic stroke patients. The association between *ALDH2* polymorphisms and cognitive impairment risk in ischemic stroke patients is shown in Table 5. At a cutoff MoCA score of 26, participants carrying the mutant *ALDH2* allele had a higher risk of cognitive impairment (OR = 3.29, 95%CI = 1.03–10.50, *p* < 0.05). Similarly, at a cutoff MoCA score of 23, participants carrying the mutant *ALDH2* allele also had a higher risk of cognitive impairment (OR = 2.65, 95%CI = 1.19–5.92, *p* < 0.05).

Further analysis of these results revealed a clear association between alcohol consumption and cognitive impairment risk in ischemic stroke patients. At a MoCA cutoff score of 26, there was an OR of 0.59 (95%CI = 0.16–0.89) in light drinkers and an OR of 1.22 (95%CI = 1.16–1.49) in excessive drinkers compared with nondrinkers. The OR values using a MoCA cutoff score of 23 were similar to the values using a cutoff score of 26.

### 3.7. ORs of Alcohol Consumption on Cognitive Impairment, Stratified by ALDH2 Polymorphism

Table 6 shows the ORs of alcohol consumption on cognitive impairment, stratified by ALDH2 polymorphism, after taking into account other potential risk factors. At a MoCA cutoff score of 26, the multivariate OR of cognitive impairment risk was 7.75 (95% CI: 1.03–113.78) for the *ALDH2* wild-type genotype in excessive drinkers compared with nondrinkers. In patients with the *ALDH2* heterozygous group, the multivariate OR (95% CI) of cognitive impairment risk compared with nondrinkers was 10.95 (1.04-114.88) in moderate drinkers. The OR values had a similar trend using a cutoff MoCA score of 23. However, in patients with the *ALDH2* heterozygous group, the multivariate OR (95% CI) of cognitive impairment risk compared with nondrinkers was 13.74 (1.96-96.51) in light drinkers, 22.36 (3.69-135.54) in moderate drinkers, and 20.93 (2.77-158.45) in excessive drinkers.

## 4. Discussion

Previous studies have revealed that *ALDH2* polymorphisms are closely related to the incidence of ischemic stroke. The current study was the first case-cohort study describing the effects of *ALDH2* polymorphisms and alcohol consumption on cognitive impairment after ischemic stroke. We demonstrated that *ALDH2* polymorphisms and alcohol consumption had a synergistic effect on cognitive impairment, even after taking other potential risk factors into account (age, gender, education, and subtype). Our results indicate that the association between alcohol consumption and cognitive impairment is stronger in the *ALDH2* heterozygous group than in the wild-type genotype group, as well as with swallowing ability.

Cognitive impairment after stroke can affect the quality of life and long-term prognosis (higher mortality and more disability) of stroke survivors [44]. Several studies have confirmed that the common functional single nucleotide polymorphism (SNP) in exon 12 of *ALDH2* is a risk indicator for ischemic stroke [21]. SNPs are the most abundant and stable genetic variations that exist in genomes [45, 46]. In the present study, the *ALDH2*∗*2* polymorphism was associated with cognitive impairment after ischemic stroke, although there was a lack of evidence of this in previous studies. Our data revealed that patients in the *ALDH2* wild-type genotype group were significantly more likely to have higher MoCA scores than patients in the mutant and heterozygous genotype group, which suggests that the *ALDH2*∗*2* polymorphism is associated with cognitive impairment after stroke.

The MoCA can be used as a dependent variable to assess the extent of early cognitive dysfunction [22]. In previous studies, cognitive impairment was defined by a MoCA cutoff score of <26 [23, 32]. However, some researchers have recommended that a cutoff score of 22/23 points might be more suitable to detect cognitive impairment [24, 31, 37]. To test the possible association between *ALDH2* polymorphisms and cognitive impairment in patients with ischemic stroke, we therefore used two different MoCA cutoff scores to investigate interaction effects. We found that the *ALDH2* mutant allele carried a higher risk of cognitive impairment using both MoCA cutoff scores. Furthermore, 4-HNE levels were higher in the cognitive impairment group than in the nonimpairment group using both MoCA cutoff scores.

Several studies have demonstrated that 4-HNE is a potential substrate for ALDH2. The levels of 4-HNE are elevated following ischemic stroke injury. Guo et al. reported that 4-HNE plays an important role in the pathogenesis of neurological diseases and is a potential biomarker for ischemic stroke [15, 16]. Our data revealed that 4-HNE levels were significantly lower in patients with the *ALDH2* wild-type genotype than in patients carrying mutant *ALDH2* alleles. Meanwhile, patients with the *ALDH2* wild-type genotype were significantly more likely to have a higher MoCA score compared with those carrying mutant alleles. This finding may explain why the *ALDH2* mutant allele carries a significantly higher risk of cognitive impairment.

Swallowing deficits are also commonly reported in patients with ischemic stroke [25]. Several studies revealed that cognitive dysfunction was associated with dysphagia [25, 26]. Therefore, we investigated the association between swallowing function and cognitive impairment using two MoCA cutoff scores. In this study, the severity of dysphagia might contribute to cognitive impairment for both MoCA cutoff scores. In addition, considering that the lesion site may affect the swallowing function, we further adopted subtypes of ischemic stroke to investigate the interaction of ALDH2 and alcohol consumption on swallowing. Our data described that both *ALDH2* genotypes showed a higher risk of dysphagia in excessive drinkers compared to nondrinkers, but the risk of dysphagia was higher in carriers of the mutant *ALDH2* allele than in noncarriers.

As an important determinant of drinking behavior, ALDH2 has a well-known role in ethanol metabolism [47]. Various longitudinal studies have reported a link between moderate alcohol consumption and improved cognitive performance [48, 49]. In our study, we also explored the association between alcohol consumption and cognitive function in patients with ischemic stroke. Considerable evidence has emerged suggesting that alcohol consumption behaviors are related to the *ALDH2*∗*2* polymorphism in Asian populations [50, 51]. We divided the ischemic stroke patients in our study into four subgroups based on their history of alcohol consumption. Alcohol consumption in the *ALDH2* wild-type genotype group was significantly higher than that in the mutant and heterozygous genotype group. This may be because carriers of *ALDH2* mutant alleles are more sensitive to alcohol, which reportedly makes them less inclined to engage in excessive drinking [52]. We used two different cognitive impairment cutoff points to analyze the association between alcohol consumption and cognitive impairment. The univariate analysis revealed a higher percentage of moderate to excessive alcohol consumption in the cognitive impairment group than in the nonimpairment group, which suggests that alcohol consumption may have an effect on cognitive impairment after ischemic stroke. We also applied a binary logistic regression model to evaluate the synergistic effects of *ALDH2* polymorphisms and alcohol consumption on cognitive impairment. Adjusted for age, gender, education, and subtype, we demonstrated that the multivariate risk of cognitive impairment was higher in excessive drinkers than in nondrinkers with the *ALDH2* wild-type genotype, while the risk of cognitive impairment was higher in light to excessive drinkers than nondrinkers with the *ALDH2* mutant or heterozygous genotype. These findings further suggest that ALDH2 might be involved in the pathogenesis and progression of cognitive impairment after ischemic stroke, as well as having a role in alcohol metabolism.

There were several limitations in the present study. First, some participants refused to participate, while blood samples were unable to be obtained from some patients; this may have caused sampling bias. Second, the MoCA scale is commonly used as a screening scale for cognitive function but fails to assess global disability after ischemic stroke. So, in this study, we also evaluated the swallowing function. The current findings are therefore considered preliminary and require validation. Third, the plasma 4-HNE levels were detected only once, at baseline, and potential fluctuations in plasma 4-HNE levels were not evaluated; we were therefore unable to adjust for this effect. Finally, although the *ALHD2*∗*2* allele is an important risk factor for ischemic stroke, results have been inconsistent over different ethnic groups, different countries, and different genders. Because the sample size was relatively small, we did not perform a stratified analysis by lesion location. We adopted subtypes of ischemic stroke to replace lesion location, and this might have affected our study results (Supplementary Table 3). To better understand the relationship between ischemic stroke impairment and *ALDH2* genotypes, future studies need to enroll larger sample sizes across multiple communities. To this end, the current study represents an ongoing effort, and we will continue to regularly update the analysis, with the aim of providing a comprehensive and easily accessible review, as well as facilitating a best-practice approach to ischemic stroke rehabilitation.

## 5. Conclusions

The present study demonstrated that *ALDH2* polymorphisms and alcohol consumption were associated with cognitive impairment and dysphagia in patients after ischemic stroke, mainly in patients with the mutant allele. ALDH2 may be involved in the pathogenesis and progression of ischemic stroke in the Han Chinese population. ALDH2 might therefore be a useful biomarker to target for cognitive rehabilitation following ischemic stroke. However, the underlying mechanisms need to be further explored.

## Figures and Tables

**Table 1:** Demographics of the study participants.

Characteristics	ALDH2 genotypes		*t*/chi square	*p* value
	∗1/∗1 (*n* = 87)	∗1/∗2 + ∗2/∗2 (*n* = 93)		
Age (years)	67.92 ± 8.02	69.88 ± 9.53	1.490	0.138
Males, *n* (%)	38 (43.7%)	49 (52.7%)	8.536	0.003
Education			7.013	0.071
<6 years	18 (20.7%)	22 (23.7%)		
6-9 years	27 (31.0%)	19 (20.4%)		
9-12 years	21 (24.1%)	37 (39.8%)		
>12 years	21 (24.1%)	15 (16.1%)		
Alcohol consumption			12.464	0.006
Nondrinkers	12.6%	21.5%		
Light drinkers	8.0%	22.6%		
Moderate drinkers	36.8%	30.1%		
Excessive drinkers	42.5%	25.8%		

Continuous variables are expressed as the mean ± standard deviation when normally distributed, and categorical variables are expressed as percentages.

**Table 2:** Baseline characteristics of ischemic stroke patients with different *ALDH2* genotypes.

Characteristics	ALDH2 genotypes		*t*	*p* value
	∗1/∗1 (*n* = 87)	∗1/∗2 + ∗2/∗2 (*n* = 93)		
BMI (kg/m^2^)	24.46 ± 2.91	25.22 ± 2.67	1.821	0.070
SBP (mmHg)	137.25 ± 20.10	142.65 ± 24.83	1.595	0.113
DBP (mmHg)	81.95 ± 9.24	82.09 ± 8.84	0.098	0.922
FPG (mmol/L)	7.31 ± 1.91	7.58 ± 1.98	0.911	0.363
TC (mmol/L)	6.16 ± 1.94	5.62 ± 1.40	2.106	0.037
TG (mmol/L)	2.13 ± 0.65	1.50 ± 0.65	1.252	0.214
HDL-C (mmol/L)	1.18 ± 0.37	1.15 ± 0.32	0.583	0.561
LDL-C (mmol/L)	2.51 ± 0.96	2.54 ± 0.75	0.234	0.815
4-HNE (ng/mL)	12.18 ± 1.94	13.42 ± 2.11	4.096	0.001

Continuous variables are expressed as the mean ± standard deviation when normally distributed, and categorical variables are expressed as percentages. BMI: body mass index; SBP: systolic blood pressure; DBP: diastolic blood pressure; FPG: fasting plasma glucose; TC: total cholesterol; TG: triglycerides; HDL-C: high-density lipoprotein cholesterol; LDL-C: low-density lipoprotein cholesterol; 4-HNE: 4-hydroxy-trans-2-nonenal.

**Table 3:** Comparison of alcohol consumption between the cognitive impairment and nonimpairment groups according to two different Montreal Cognitive Assessment (MoCA) cutoff scores.

Alcohol consumption	Method 1		Method 2	
	Impairment	Nonimpairment	Impairment	Nonimpairment
Nondrinkers	21 (13.4%)	10 (43.5%)	12 (9.9%)	19 (32.2%)
Light drinkers	24 (15.3%)	4 (17.4%)	18 (14.9%)	10 (16.9%)
Moderate drinkers	52 (33.1%)	8 (34.8%)	44 (36.4%)	16 (27.1%)
Excessive drinkers	60 (38.2%)	1 (4.3%)	47 (38.8%)	14 (23.7%)
Chi square	17.419		15.238	
*p* value	0.001		0.002	

Method 1: MoCA cutoff score of 26. Method 2: MoCA cutoff score of 23.

**Table 4:** Comparison of swallowing function between the cognitive impairment and nonimpairment groups according to two different MoCA cutoff scores.

Swallowing	Method 1		*p*	Method 2		*p*
	Impairment	Nonimpairment		Impairment	Nonimpairment	
Level I	9 (5.7%)	23 (100.0%)	0.001	0 (0.0%)	32 (54.2%)	0.001
Level II	44 (28.0%)	0 (0.0%)		17 (14.0%)	27 (45.8%)	
Level III	53 (33.8%)	0 (0.0%)		53 (43.8%)	0 (0.0%)	
Level IV	29 (18.5%)	0 (0.0%)		29 (24.0%)	0 (0.0%)	
Level V	22 (14.0%)	0 (0.0%)		22 (18.2%)	0 (0.0%)	

**Table 5:** Adjusted odds ratios (95% confidence intervals)^a^ for the separate effects of *ALDH2* polymorphism and alcohol consumption on cognitive impairment in ischemic stroke patients.

	Method 1	Method 2
ALDH2 genotypes		
∗1/∗1	Ref	Ref
∗1/∗2 + ∗2/∗2	3.29 (1.03-10.50)^∗^	2.65 (1.19-5.92)^∗^
Alcohol consumption		
Nondrinkers	Ref	Ref
Light drinkers	0.59 (0.16-0.89)^#^	0.68 (0.33-0.89)^#^
Moderate drinkers	0.77 (0.21-1.06)	1.08 (0.83-2.19)
Excessive drinkers	1.22 (1.16-1.49)^#^	1.78 (1.03-2.15)^#^

^a^Models were adjusted for age, gender, and education. ^∗^*p* < 0.05 vs. ALDH2 wild-type genotype group (ALDH2∗1/∗1). ^#^*p* < 0.05 vs. nondrinkers.

**Table 6:** Multivariate odds ratios (95% confidence intervals)^a^ for alcohol consumption on cognitive impairment in stroke patients, stratified by *ALDH2* polymorphism.

ALDH2 genotypes	Alcohol consumption	Method 1	Method 2
∗1/∗1	Nondrinkers	Ref	Ref
	Light drinkers	1.75 (0.20-15.19)	0.45 (0.05-3.91)
	Moderate drinkers	2.97 (0.60-14.68)	2.43 (0.54-11.02)
	Excessive drinkers	7.75 (1.03-113.78)^∗^	3.93 (0.84-18.44)

∗1/∗2	Nondrinkers	Ref	Ref
	Light drinkers	15.99 (0.96-266.02)	13.74 (1.96-96.51)^#^
	Moderate drinkers	10.95 (1.04-114.88)^#^	22.36 (3.69-135.54)^#^
	Excessive drinkers	—	20.93 (2.77-158.45)^#^

∗2/∗2	Nondrinkers	—	—
	Light drinkers	—	—
	Moderate drinkers	—	—
	Excessive drinkers	—	—

^a^Models were adjusted for age, gender, education, and subtype. ^∗^*p* < 0.05 vs. nondrinkers with ALDH2 wild-type genotype (ALDH2∗1/∗1). ^#^*p* < 0.05 vs. nondrinkers with ALDH2 mutant genotype (ALDH2∗1/∗2).

## Data Availability

The datasets used and/or analyzed during the current study are available from the corresponding author on reasonable request.

## Supplementary Materials

Supplementary Materials Supplementary Table 1: comparison of MoCA subscores based on ALDH2 polymorphisms and alcohol consumption. Supplementary Table 2: comparison of MoCA subscores based on ALDH2 polymorphisms and alcohol consumption. Supplementary Table 3: multivariate odds ratios (95% confidence intervals) for alcohol consumption on swallowing ability in stroke patients, stratified by ALDH2 polymorphism.
