# Supplementary material for: Synergistic Effects of Aldehyde Dehydrogenase 2 Polymorphisms and Alcohol Consumption on Cognitive Impairment after Ischemic Stroke in Han Chinese
Source: Behav Neurol. 2021 Jun 24;2021:6696806. doi: 10.1155/2021/6696806 (PMC8253650; doi:10.1155/2021/6696806)
Supplement: Supplementary Materials — Supplementary Table 1: comparison of MoCA subscores based on ALDH2 polymorphisms and alcohol consumption. Supplementary Table 2: comparison of MoCA subscores based on ALDH2 polymorphisms and alcohol consumption. Supplementary Table 3: multivariate odds ratios (95% confidence intervals) for alcohol consumption on swallowing ability in stroke patients, stratified by ALDH2 polymorphism. [file 6696806.f1.docx]

Supplementary Table 1 Comparison of alcohol consumption between

two *ALDH2* genotypes by gender

| Alcohol Consumption | Male | | Female | |
| --- | --- | --- | --- | --- |
|  | *1/*1 | *1/*2+*2/*2 | *1/*1 | *1/*2+*2/*2 |
| Non-drinkers | 6（15.8%） | 8（16.3%） | 5（10.2%） | 12（27.3%） |
| Light drinkers | 3（7.9%） | 9（18.4%） | 4（8.2%） | 12（27.3%） |
| Moderate drinkers | 18（47.4%） | 12（24.5%） | 14（28.6%） | 16（36.4%） |
| Excessive drinkers | 11（28.9%） | 20（40.8%） | 26（53.1%） | 4（9.1%） |

Supplementary Table 2: Comparison of MoCA subscores based on *ALDH2* polymorphisms

and alcohol consumption

| Subscore  of MoCA | *1/*1 | | | |  | *1/*2+*2/*2 | | | |  |
| --- | --- | --- | --- | --- | --- | --- | --- | --- | --- | --- |
|  | Non-drinkers | Light drinkers | Moderate drinkers | Excessive drinkers | *p* | Non-drinkers | Light drinkers | Moderate drinkers | Excessive drinkers | *p* |
| Visuospatial  /Executive | 3.27±1.56 | 2.86±1.77 | 2.94±1.66 | 3.43±1.30 | 0.533 | 3.50±1.76 | 2.90±1.51 | 3.46±1.29 | 3.67±1.34 | 0.348 |
| Naming | 3.00±0.00 | 3.00±0.00 | 2.97±0.18 | 2.92±0.49 | 0.856 | 2.90±0.45 | 3.00±0.00 | 3.00±0.00 | 2.88±0.61 | 0.536 |
| Attention | 5.09±1.51 | 4.14±2.27 | 4.28±1.82 | 4.35±1.96 | 0.625 | 4.95±1.61 | 4.43±1.94 | 4.93±1.21 | 4.50±1.87 | 0.595 |
| Language | 2.97±1.15 | 1.84±1.07 | 1.82±1.25 | 1.43±1.13 | 0.003 | 2.43±0.63 | 1.85±0.94 | 1.75±0.61 | 1.76±0.64 | 0.005 |
| Abstraction | 3.64±1.21 | 4.00±0.00 | 3.25±1.41 | 3.68±1.00 | 0.309 | 3.60±1.05 | 3.86±0.48 | 3.64±0.78 | 3.83±0.82 | 0.620 |
| Delayed recall | 1.93±0.74 | 1.56±0.66 | 1.35±0.64 | 1.29±0.38 | 0.010 | 2.21±0.84 | 1.93±0.76 | 1.76±0.76 | 1.50±0.47 | 0.013 |
| Orientation | 3.36±0.81 | 3.43±0.79 | 2.97±1.28 | 3.41±0.96 | 0.349 | 3.50±0.95 | 3.33±1.11 | 3.64±0.62 | 3.58±0.83 | 0.651 |

Supplementary Table 3: Multivariate odd ratios (95% confidence intervals) ^a^ for alcohol consumption on swallowing ability in stroke patients, stratified by *ALDH2* polymorphism.

| ALDH2  Genotypes | Alcohol  consumption | odd ratios  (95% confidence intervals) |
| --- | --- | --- |
| *1/*1 | Non-drinkers | Ref |
|  | Light drinkers | 1.37 (0.17-11.01) |
|  | Moderate drinkers | 3.32 (0.68-16.27) |
|  | Excessive drinkers | 11.99 (1.91-75.21) ^*^ |
| *1/*2 | Non-drinkers | Ref |
|  | Light drinkers | 18.09 (1.76-185.51) ^#^ |
|  | Moderate drinkers | 81.51(2.50-200.33) ^#^ |
|  | Excessive drinkers | —— |
| *2/*2 | Non-drinkers | —— |
|  | Light drinkers | —— |
|  | Moderate drinkers | —— |
|  | Excessive drinkers | —— |

^a^ Models were adjusted for age, gender, education and subtype

^*^ *p* < 0.05 vs. non-drinkers with *ALDH2* wild-type genotype (*ALDH2*1/*1*)

^#^ *p* < 0.05 vs. non-drinkers with *ALDH2* mutant genotype (*ALDH2*1/*2*)
